# Mapping the Scientific Landscape of Diabetes Research in Malaysia (2000–2018): A Systematic Scientometrics Study

**DOI:** 10.3390/ijerph18010318

**Published:** 2021-01-04

**Authors:** Kurubaran Ganasegeran, Chee Peng Hor, Mohd Fadzly Amar Jamil, Purnima Devi Suppiah, Juliana Mohd Noor, Norshahida Abdul Hamid, Deik Roy Chuan, Mohd Rizal Abdul Manaf, Alan Swee Hock Ch’ng, Irene Looi

**Affiliations:** 1 Clinical Research Center, Seberang Jaya Hospital, Ministry of Health Malaysia, Penang 13700, Malaysia; cheepengh@yahoo.com (C.P.H.); fadzly.crc@gmail.com (M.F.A.J.); purnima.crc@gmail.com (P.D.S.); juliana_crc@yahoo.com (J.M.N.); norshahida.crc@gmail.com (N.A.H.); deikroy@hotmail.com (D.R.C.); alanchng1978@gmail.com (A.S.H.C.); irenelooi@yahoo.com (I.L.); 2 Department of Medicine, Kepala Batas Hospital, Penang 13200, Malaysia; 3 Institute for Clinical Research, National Institutes of Health, Selangor 40170, Malaysia; 4 Department of Community Health, Faculty of Medicine, Universiti Kebangsaan Malaysia, Kuala Lumpur 56000, Malaysia; 5 Medical Department, Seberang Jaya Hospital, Penang 13700, Malaysia

**Keywords:** scientometrics, diabetes mellitus, scientific landscape, science mapping, Malaysia

## Abstract

The escalated burden of diabetes on the population’s health has catalyzed rigorous scientific research to produce appropriate evidence for treatment and control. Malaysia suffers from the leading diabetes epidemic within the Western Pacific region. It is crucial to map the scientific landscape of diabetes research for the country to identify trends in productivity and determine whether research efforts are directed toward the needs-gaps priority for evidence synthesis that could be used for the drafting of policies and guidelines. This systematic scientometrics study was conducted to map the scientific research output (trends and distribution, citation frequency, keywords link visualization, and thematic cluster conceptualization) related to diabetes between 2000–2018 in Malaysia. Using three international databases (PubMed, EMBASE, Scopus) and one local database (MyCite), scientific publication records related to diabetes in Malaysia between 2000 and 2018 were retrieved and analyzed using quantitative and qualitative methodologies. Microsoft Excel 2016, EndNote X9.2, BibExcel 2016, GraphPad Prism 8.0.1, VOS viewer software 1.6.13, and R software version 1.3.959 were used to analyze the trend and contents of diabetes publications. A total of 2094 publication records that accounted for 35,497 citations were analyzed. Kuala Lumpur was the most scientifically productive state in Malaysia, contributing 754 papers. *Medical Journal of Malaysia* had the highest number of publications. The inflection point of the Malaysian diabetes research output was in 2013, with most publications being non-collaborative research works. Most publications originated from academia, especially from local public universities. The overall publication productivity of diabetes research in Malaysia was conceptualized into eleven thematic clusters, with clinical and animal studies being the most prevalent themes. The diabetes literature in Malaysia has grown steadily over the past 19 years. However, the cumulative evidence remains inadequate and is insufficiently powered to guide policymaking and the control of diabetes. It does not yet seem feasible to direct the diabetes epidemic curve to a plateau for the Malaysian population based on Malaysian diabetes publications.

## 1. Introduction

Population aging and the epidemiological transition toward a sedentary lifestyle have escalated the burden of diabetes worldwide. Approximately 463 million adults had type 2 diabetes worldwide as of 2019, with 79% of them originating from low- and middle- income countries (LMICs) [1]. While this figure is projected to show a continued rise in the coming years, the prevalence of gestational diabetes (GDM) among pregnant mothers has concomitantly exhibited a rising trend within the past few decades, collectively reporting around 30% of cases across LMICs [2]. In a recent meta-analysis, the global prevalence of type 1 diabetes was reported to be approximately 9.5% [3]. The Southeast Asian (SEA) region reported the highest prevalence of diabetes (8.8%) [4], and Malaysia topped the Western Pacific region, affecting almost 3.65 million people (16.8%) of the total 21.71 million adult Malaysian population [5]. Populations with high rates of diabetes often include many people suffering significant disabilities, reduced quality of life, and increased mortality, and create greater economic burdens on governments and societies [6]. With diabetes affecting every stratum of the population and every region, healthcare systems worldwide continuously struggle to combat the diabetes epidemic through public health control measures and newer treatment initiatives. Such efforts will be effective and efficacious when newer reliable evidence is yielded through continuous scientific research, which is often reported through scholarly publications for adoption into real-life practice.

The level of scientific research activities varies significantly across countries, as the ability to generate efficient science powerhouses is highly dependent on the capacity for harnessing the available human capital and financial resources of a nation [7]. However, assessing the results produced through scientific research works is highly crucial and important to drive the country’s healthcare system and people’s health-related states forward. Assessing the results produced by scientific works available through scholarly publishing requires the capacity to study the “science of science” [8] for a given health-related state. This novel approach, collectively termed “scientometrics,” aims to provide a systematic instrumental approach to utilizing various statistical approaches and synthetic indicators to quantify scientific outputs that are principally available through published articles, citations, patents, authorships, or the impact of the output [9]. Scientometrics may be categorized into microscientometrics (assessment of authors, researchers, journals, and institutions) or macroscientometrics (assessment of scientific output in large numbers at the national or regional levels, such as indicators showing the number of articles, publications in journals, or the number of citations) [9]. Although scientometric studies at the country level are capable of detecting the mechanisms that generate greater or lesser scientific output quantitatively, their qualitative assessments are sufficiently robust for the entire scientific enterprise, ranging from the evaluation of funding applications to the reputation of scientists to the departments or institutes that have the capacity to build collaborative strengths and networks [9].

Scientometric methodologies have the capacity to evaluate the sociological phenomena related to scientific communities. Such methodologies have been used to determine the impact of research and map scientific networks while being able to monitor the trends and evolution within scientific fields [10]. They have been widely used to assist researchers in appraising how research communities are trending or evolving through reflections of scientific publications [11] and in specific disease fields, such as diabetes [12,13,14]. This literature metrology system is increasingly popular for its application toward drafting a country’s healthcare policy or clinical practice guidelines.

As Malaysia’s diabetes burden is burgeoning, it is important to execute a scientometric evaluation on the context of diabetes to inform current trends of scientific-related works and the potential needs-gaps to refocus the nation’s diabetes research field across specific themes. Such efforts would yield timely and relevant evidence that would be useful for public health interventions, policy drafting, and routine clinical practice in the quest to control the diabetes epidemic. The current study aimed to map the entire diabetes research landscape between 2000 and 2018 in Malaysia, both quantitatively and qualitatively (trends and distribution, citation frequency, keywords link visualization, and thematic cluster conceptualization), through a scientometric approach.

## 2. Materials and Methods

### 2.1. Data Sources and Search Strategy

**Figure 1:**
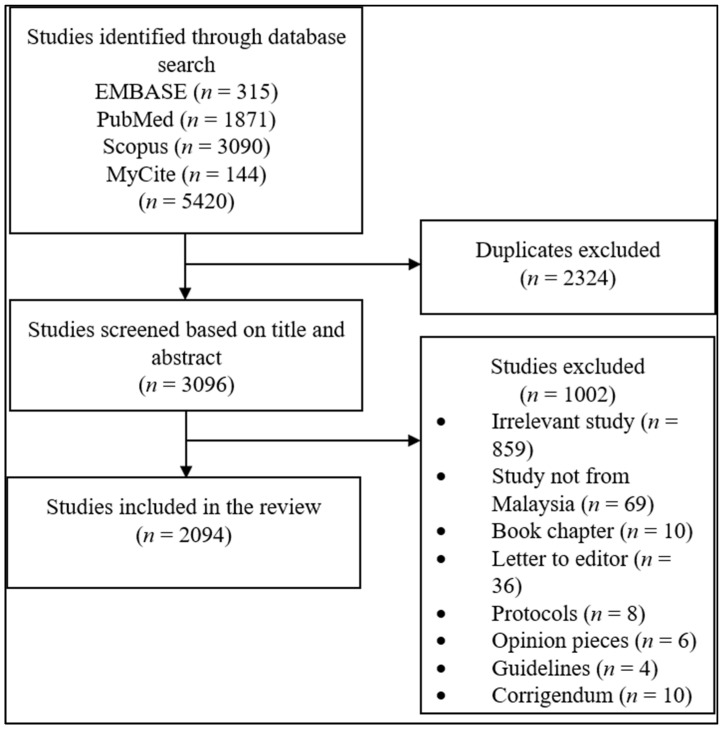
Study flowchart of the data retrieval process.

This systematic scientometrics investigation evaluated the entire scientific literature on diabetes published in Malaysia between January 2000 and December 2018. We explored and retrieved the literature of the diabetes research output across three major international databases, namely SCOPUS, PubMed, and EMBASE, and one local database (MyCite) using keywords (diabetes OR diabetology OR diabetic OR diabetes mellitus OR type 1 diabetes OR insulin-dependent diabetes OR type 2 diabetes OR non-insulin-dependent diabetes OR gestational diabetes) AND publishing year = (2000–2018) AND affiliation = (Malaysia). The complete retrieval process is exhibited in Figure 1.

### 2.2. Data Extraction

The references identified from the four databases were imported into EndNote X9.2. All searches were conducted on a single day to avoid changes in the number of publications and citations as much as possible. Publications were restricted to original research articles, case reports, and review papers only. Duplicate removal was managed in EndNote through the “find duplicates” function. After the removal of duplicates, the remaining references in EndNote were normalized using the RefMan (RIS) Export feature and subsequently imported to BibExcel software (Olle Persson, version 2016-02-20). BibExcel automates the extraction of the fundamental characteristics of each article as follows: TI (title of the article), AU (author), PY (publication year), T2 (journal name), and AD (affiliation of the authors). Two researchers independently verified all eligible publications of the diabetes research yield for Malaysia. The verification of additional data retrieval was conducted manually by two independent researchers. These data included authors’ affiliation type (government agency, private healthcare, local public university, local private university, or Ministry of Health), journal type (local or international journal), collaboration works (no collaboration, national level collaboration, or international level collaboration), and the number of citations. Any differences between the two independent researchers were discussed with a third researcher to reach a consensus.

### 2.3. Statistical Analysis

The finalized data in the database were imported into Microsoft Office Excel 2016 (Microsoft Corporation, Washington, DC, USA), GraphPad Prism version 8.0.1 (GraphPad Software Inc, San Diego, CA, USA), VOS viewer version 1.6.13 (Centre of Science and Technology Studies of Leiden University, Leiden, Netherlands), and R version 1.3.959 (RStudio, PBC, Boston, MA, USA) to execute both the quantitative and qualitative analyses. Microsoft Office Excel was used to analyze the characteristics of the publications, including the authors’ affiliation, citation frequency, collaboration frequency, journal type, and study designs. GraphPad Prism software was used to generate the time trend of publications through a logistic growth curve. The application uses the Marquardt and Levenberg method of nonlinear least-squares regression for estimating the parameters of logistic curves, with no assumption of saturation level provided [15]. Given its sensitiveness and ability to predict future trends in the literature [16], the logistic growth curve fitting formula $f\left(x\right)=c/\left[1+ae^{-b\left(x-2000\right)}\right]$ was used to model the cumulative volume of articles published by year. The symbol *x* represents the year, while *f*(*x*) was the cumulative volume of publications by year. The point in time when the publication growth rate moved from positive to negative was the inflection point of the logistic growth curve, which is generated through the formula T=ln (a/b). The goodness of fit of the logistic growth model was determined through the yielded *R* squared (*R*^2^) value. *R*^2^ is a fraction of all the variance in *f*(*x*) explained by the model, with values ranging between 0 and 1. If *R*^2^ equals 1.0, then each *f*(*x*) value is predicted perfectly by the model, with no random variability. If *R*^2^ equals 0.0, the model’s prediction of *f*(*x*) is extremely poor.

A Lotka’s law curve was generated to explore the relationships between the number of publications and the number of authors published within the scientific landscape of diabetes research yield in Malaysia. Hotspot analysis of scientific productivity values based on per million population according to states in Malaysia was analyzed through a generated heat map using R software (packages: ggplot2, maptools, rgeos, Cairo, ggmap, scales, RColorBrewer, rgdal, raster) with set.seed (8000). VOS viewer software was used to generate radar charts for visualizing the network of co-authorships and keyword links of the diabetes research output. Frequent keyword occurrences and thematic analyses of the identified clusters were conceptualized. A two-dimensional radar chart that conceptually provided a framework for mapping and clustering keywords’ co-occurrence was built using the VOS viewer software [17]. The keywords were mapped in such a way that their relatedness was associated with proximity on the map. The size of each node reflects that keyword’s frequency, while the weight of each connecting line indicates the number of publications in which the connected keywords co-occur. The keyword clusters were synthesized based on the modularity-clustering approach, whereby keywords are placed in a cluster based on the frequency of co-occurrence, signified by colors [17]. The conceptualization of the identified clusters was aimed toward reflecting the most prevalent themes. Common themes were identified using both open and axial coding principles, as advocated for by the grounded theory approach [18]. For the radar chart that identified network co-authorships, the size of the circle represents the density of the output productivity, while the thickness of the connecting lines represents the collaboration strength.

## 3. Results

### 3.1. Evaluation of Malaysia’s Diabetes Research Productivity

#### 3.1.1. Trend Analysis

**Figure 2:**
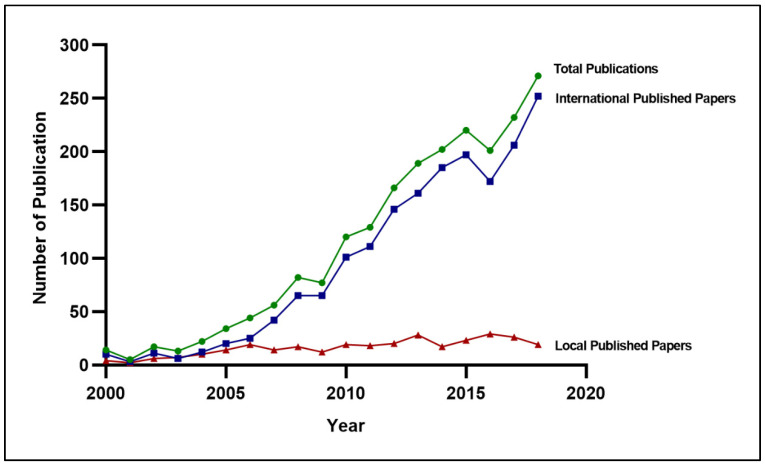
Trend of diabetes research productivity from 2000–2018 in Malaysia (*n* = 2094).

From 2000 to 2018, Malaysia contributed to 2094 scientific research papers related to diabetes mellitus within the scholarly literature (Table S1: Supplementary information of papers included). The trend of total scientific productivity increased steadily over time between the years 2000 and 2018. Similar publication trends were observed across the international scientific landscape. In contrast, publications in local journals were low and static over the past 19 years (Figure 2).

#### 3.1.2. Diabetes Research Productivity over Time

**Figure 3:**
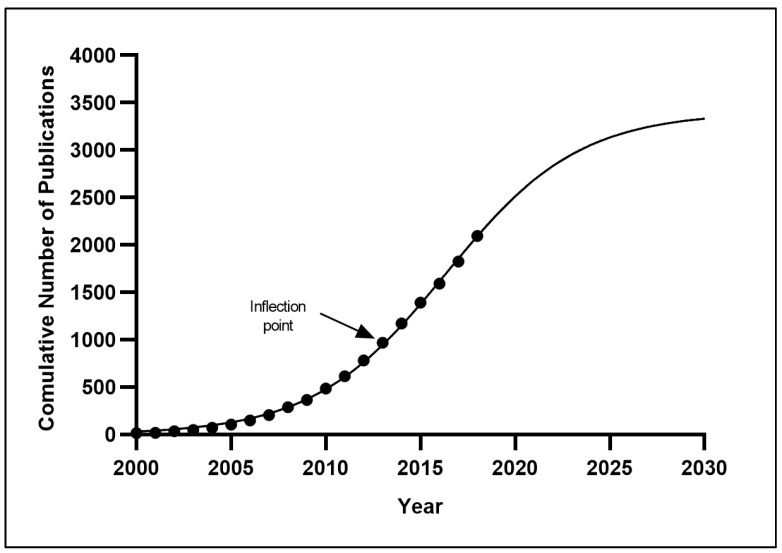
Logistic model fitting curve of the growth trends of the diabetes research productivity in Malaysia (2000–2018, with a projection till 2030).

A logistic growth curve was constructed to analyze the time curve of the cumulative number of diabetes research papers from Malaysia, and the inflection point (the point that the growth rate of the number of research output changed from positive to negative) was derived to predict future trends of diabetes-related research publications from Malaysia. Figure 3 shows the model fitting curve for Malaysia’s diabetes publication growth trends. The inflection point of Malaysia’s diabetes research publications occurred in 2013, as derived from the logistic growth curve. The *R*^2^ goodness of fit of the model growth was 0.9638, confirming that the cumulative number of publications predicted a nearly perfect growth model, with no random variability. It is projected that the diabetes research works from Malaysia will continue to rise at a fast pace within the next ten years, as a saturation point (the level of curve reaching a plateau phase) was not observed.

#### 3.1.3. Frequency Analysis

A significant portion of the scientific papers produced in Malaysia had no collaborations with other scientists or researchers from different institutions (833 papers, 39.8%). The highest number of publications was observed in the year 2018. The publications of collaborative works with international scientists and researchers accounted for approximately 655 papers or 31.3% of the total publications. The highest number of such publications was observed in the year 2018. Publications of collaborative works with local scientists and researchers accounted for 606 papers or 28.9% of the total scientific productivity. The highest number of such publications was observed in the year 2015 (Table 1).

#### 3.1.4. Distributional Hotspot Analysis

**Figure 4:**
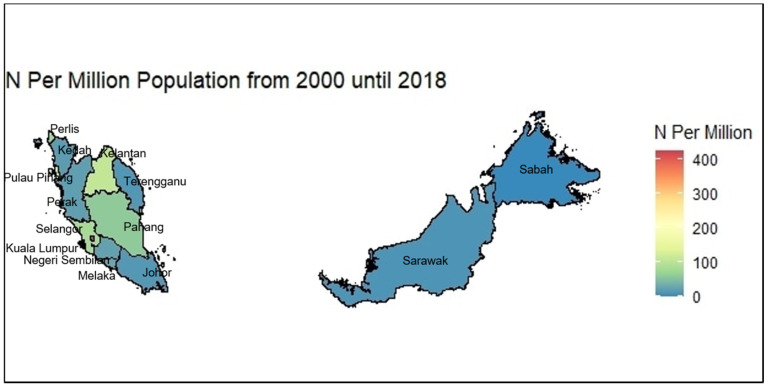
Hotspot analysis of the scientific productivity values by states in Malaysia.

Hotspot analysis on the number of publications across states in Malaysia found the top five states that produced the highest number of scientific publications over time were Kuala Lumpur (754 papers), Selangor (537 papers), Kelantan (203 papers), Pulau Pinang (190 papers), and Pahang (102 papers). The state with the lowest publication output was Sabah (9 papers). Figure 4 shows the distribution of scientific publications per million population across states in Malaysia. When comparing the adequacy of scientific productivity per million population across states in Malaysia, Kuala Lumpur appeared to be the most scientifically productive state (productivity value = 423.43), followed by Putrajaya (231.21), Kelantan (107.65), Pulau Pinang (107.07), Selangor (82.26), Perlis (70.75), Pahang (60.91), Melaka (27.94), Negeri Sembilan (23.89), Perak (22.29), Kedah (18.80), Johor (15.14), Terengganu (14.45), and Sarawak (11.38). The state with the lowest scientific productivity value was Sabah (2.31) (Figure 4).

### 3.2. Citation Analyses of Malaysia’s Diabetes Research Productivity

#### 3.2.1. Citation Trend

**Figure 5:**
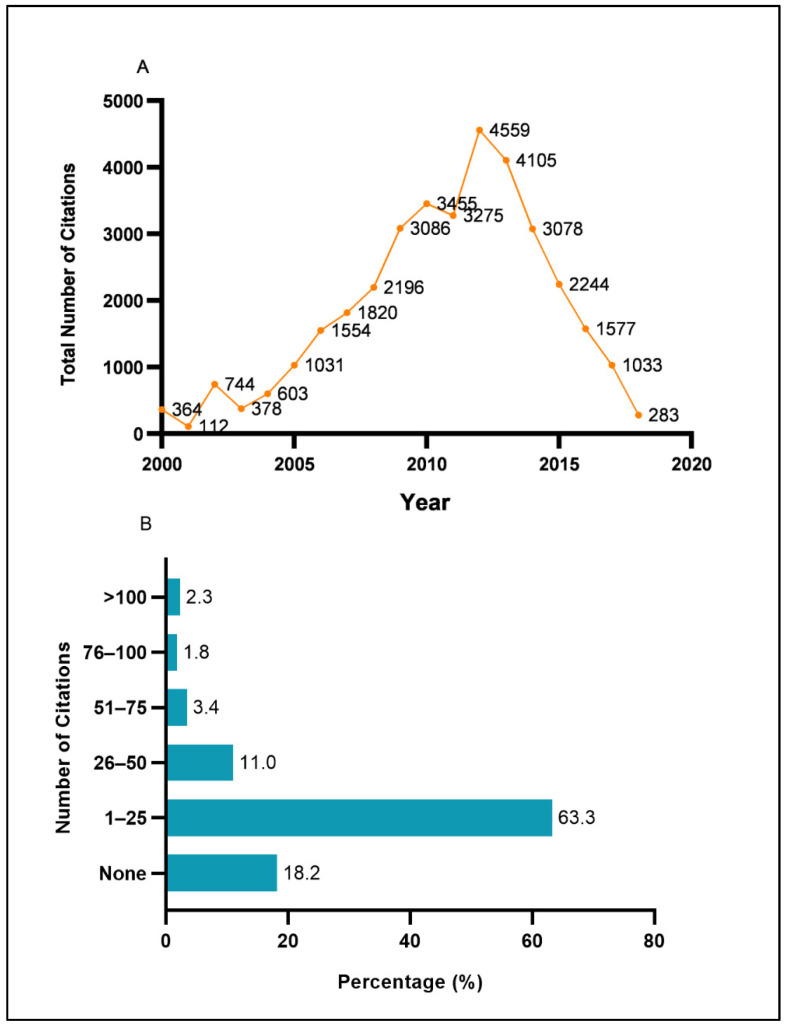
Citations analysis: (**A**) trend of the citations from 2000 to 2018 and (**B**) frequency of citations by range.

Figure 5A shows the citation trends overtime for Malaysia’s diabetes research productivity. There was an overall increasing trend of total citations between 2000 and 2012 with intermittent dips of total citations in 2001, 2003, and 2011. The peak of the total number of citations of Malaysia’s diabetes scientific research productivity occurred in 2012 (4559 citations).

#### 3.2.2. Citation Frequency of the Identified Papers

Of the 2094 papers identified, a cumulative frequency of 35,497 citations of those papers were identified. The average cited frequency per publication was 16.95 times. The average annual citation of all papers was 1868.26 times, while the average annual citation per paper was 0.9 times. The most-cited paper was identified in the year 2009 with 947 citations to date. The top ten most-cited papers are shown in Table 2. Almost 63.3% of the total 2094 papers published generated 1–25 citations. About 2.3% of the total papers had more than 100 citations, while 18.2% of them have not received a single citation (Figure 5B).

The citations of publications that were not collaborative works with scientists and researchers from other institutions topped the scientific publishing landscape and accounted for 13,425 citations or 37.8% of the total citations. The highest number of non-collaborative work citations was observed in the year 2012. The citations of publications that were of collaborative works with international scientists and researchers accounted for approximately 13,375 citations or 37.7% of the total citations. The highest number of international collaborative work citations was observed in the year 2013. The citations of publications of collaborative works with local scientists and researchers accounted for 8697 citations or 24.5% of the total citations. The highest number of national collaborative work citations was observed in the year 2014 (Table 1).

### 3.3. Source Evaluation of Malaysia’s Diabetes Research Productivity

#### 3.3.1. Highly Contributing Journals

The 2094 papers published between 2000 and 2018 were accommodated in 807 peer-reviewed journals. The top 10 journals for diabetes research productivity in Malaysia are exhibited in Table 3. *Medical Journal of Malaysia* published the most (95 papers) and *Institute of Electrical and Electronics Engineers (IEEE)* ranked second (68 papers).

#### 3.3.2. Highly Contributing Institutions

**Figure 6:**
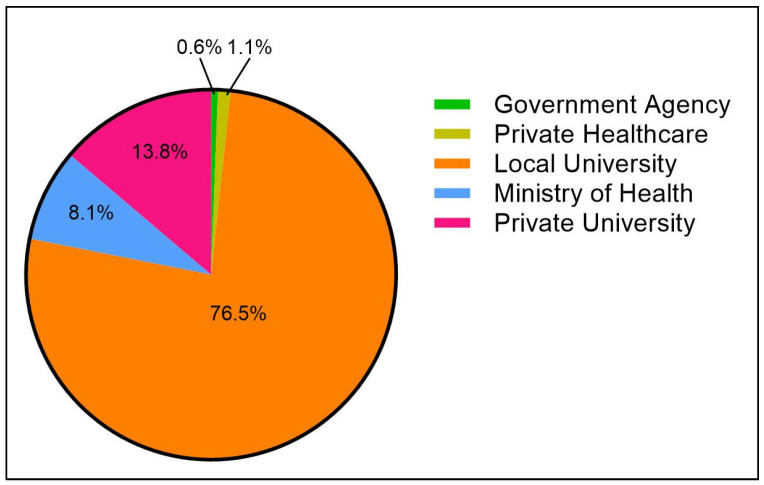
Contributing institutions toward diabetes research productivity.

The bulk of the diabetes research publications were contributed by academia, of which 1602 papers (76.5%) were contributions made by local public universities and 289 papers (13.8%) came from local private universities. The third-highest contributor to the diabetes scholarly literature in Malaysia originated from a service-oriented institution, with the Ministry of Health, Malaysia, facilities contributing to approximately 170 papers or 8.1% of the total output published between 2000 and 2018 (Figure 6). The institutions that contributed to the top 10 most-cited papers are exhibited in Table 2.

#### 3.3.3. Highly Contributing Author-Researchers

**Figure 7:**
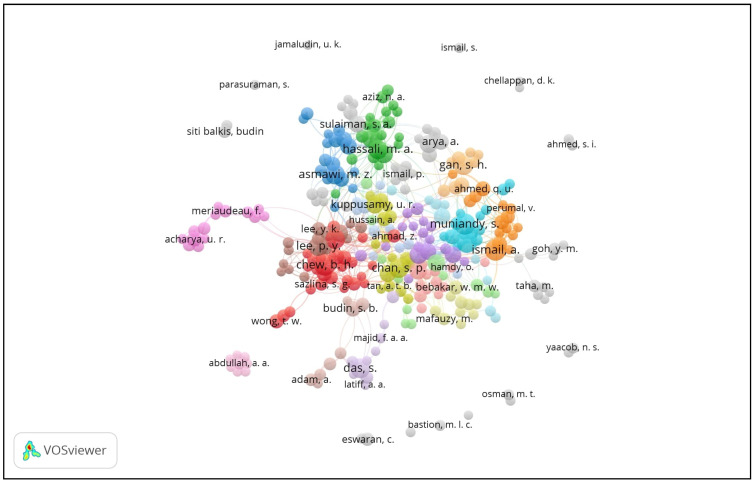
Radar chart showing the network of co-authorships of the diabetes research output. Note: The threshold was set at ≥5 publications. The size of the circle represents the density of the output productivity, while the thickness of the connecting lines represents the collaboration strength.

The top five contributing author-researchers of diabetes-related research publications between 2000 and 2018 were Muniandy, S. (35 publications), Chew, B.H., and Ismail, A. (33 publications each), Ismail, I.S. (32 publications), and Lee, P.Y. (29 publications). The stratified analyses of the top five authors with the strongest collaborative link strengths were Lee, P.Y. (link strength of 120), Chew, B.H. (link strength of 113), Ismail, I.S. (link strength of 98), Cheong, A.T. (link strength of 75) and Ismail, A. (link strength of 74). The radar chart in Figure 7 exhibits the authors’ collaborative link visualization across the diabetes scientific works in Malaysia.

#### 3.3.4. Comparison of the Author-Researcher Contributions and the Needs-Demand Analysis

**Figure 8:**
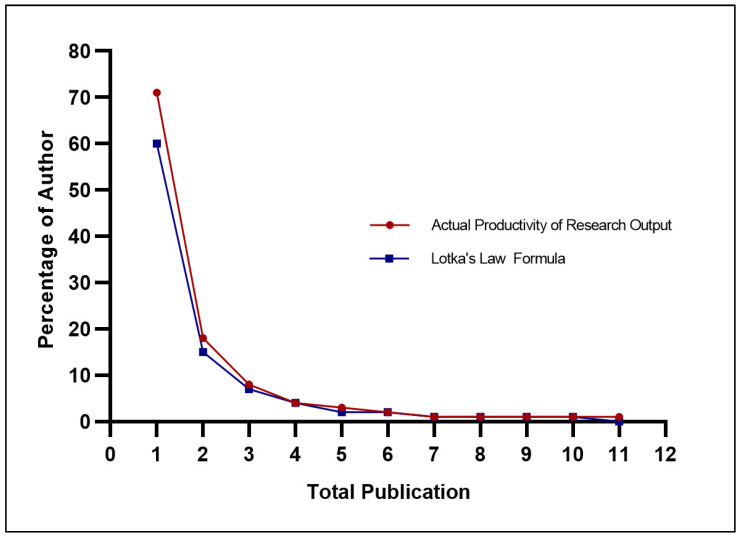
Percentage of authors publishing a certain number of diabetes-related papers in Malaysia compared with Lotka’s law.

To determine whether the diabetes research productivity patterns in Malaysia were consistent with the authors’ availability and the needs-demand of the diabetes burden, we produced a Lotka’s law analytic curve (Figure 8). Lotka’s law indicates an inverse relationship between the number of publications and the number of authors producing those publications. For all authors in a given field, the rule proposed that 60% of authors will have produced only one publication, 15% will have produced two publications, 7% will have produced three publications, and about 6% will have produced ten publications. Figure 8 shows that the diabetes research output generated in Malaysia was nearly consistent with the scientific productivity curve, as proposed by Lotka’s law.

### 3.4. Content Evaluation of Malaysia’s Diabetes Research Productivity

#### 3.4.1. Document Analyses of the Study Types and Designs

**Figure 9:**
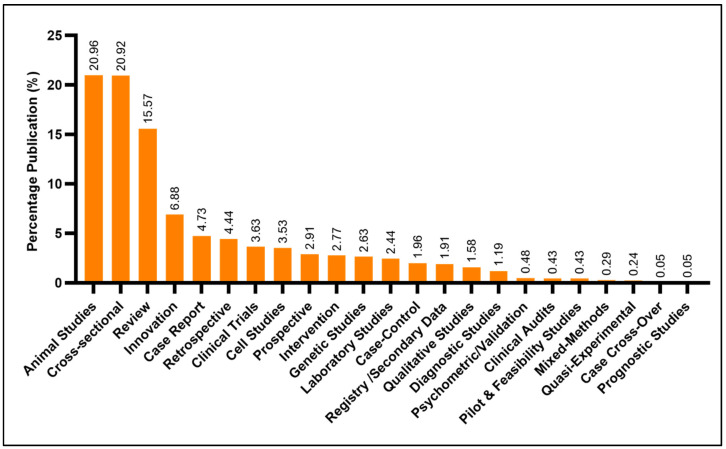
Common study types and designs of diabetes-related research from Malaysia.

The majority of the total publications were animal studies and cross-sectional in nature, accounting for approximately 21% each. Review papers accounted for 15.6%, followed by innovation studies and patient case reports (6.9% and 4.7%, respectively). Case cross-overs and prognostic studies were the least frequently produced works and accounted for less than 1% of the total diabetes scientific literature produced in Malaysia (Figure 9).

#### 3.4.2. Keyword Links Visualization and Thematic Cluster Analyses

**Figure 10:**
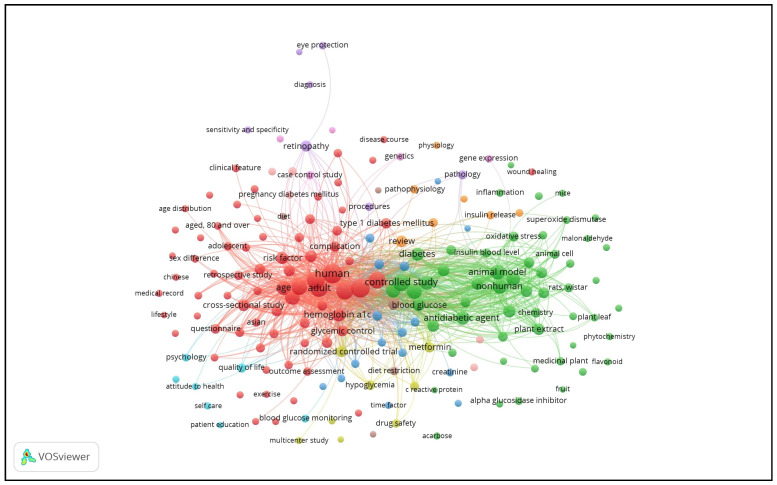
Radar chart showing the keyword links visualization of the diabetes research output. Note: The threshold was set at ≥35 occurrences. Keywords shown in the same color are closely related and clustered together. The size of nodes and words represent weights; the bigger the nodes and words, the larger the weights. The distance between two nodes reflects the strengths between them; a shorter distance means stronger relations. The line between two keywords means that they appeared together; the thicker the line, the higher the co-occurrence.

Figure 10 shows the keywords links visualization of the diabetes research output. The network of the keyword links visualization generated eleven major thematic clusters, with clinical studies as the largest cluster with 75 items, followed by animal studies (47 items), co-morbidities (9 items), drug studies (9 items), biomarker studies (8 items), complications (8 items), treatment (8 items), control measures (8 items), eye complications (6 items), health outcomes, knowledge, attitudes, and practice (6 items), and gestational diabetes (5 items) (Table 4).

## 4. Discussion

To the authors’ knowledge, this study is the first scientometric study to characterize the scientific publications of diabetes-related research in Malaysia over nearly two decades. The continuous growth of publications has demonstrated an increased interest in diabetes and efforts from the local scientific community. Such a trend was consistent with the growth patterns of diabetes-related research activity globally [12,19,20,21,22,23,24]. Nonetheless, Malaysia has yet to achieve the milestone of being in the top 25 leading countries for diabetes research in the world [25], despite being one of the Asian countries to be the hardest hit by the epidemic.

Malaysia has seen an increasing disease burden of diabetes over the past decades. The prevalence of diabetes among Malaysian adults had increased from 8.3% in 1996 to 14.9% in 2006, and subsequently to 18.3% in 2019 [26,27]. It is projected to nearly double to 31.3% of the 36.02 million population by 2025 [28,29]. The diabetes epidemic has caused enormous socio-economic impacts on the population. The current scientific literature has yet to adequately reflect the Malaysian health system, policies, and strategies. There is an urgent need to upscale research activities and produce evidence to better understand the needs and gaps of diabetes care, ranging from preventive measures to diabetes control and impact reduction strategies.

### 4.1. Diabetes Research Productivity

The research productivity measured by the annual number of publications showed a steep 18-fold increase from 14 in 2000, up to 271 in 2018. The average yearly publication was 110.2, higher than Iran (6.19) [12], Cuba (33.9) [30], and India (4.82) [17]. The annual growth rate of publications in Malaysia was 27.5%, higher than that of Cuba (8.3%), India (13.7%), and Iran (25.5%). Driven by top national priorities and the population’s needs [31], this body of research evidence is projected to grow with a cumulative estimate of 3332 publications by 2030. The majority of the scientific works were published in international journals, while diabetes-related publications in local journals had plateaued. Factors, such as impact factors, wider journal readership, incentive-driven publication policies, and international peers’ recognition were driving forces for Malaysian researchers to publish in international peer-reviewed journals [32]. In addition, Malaysian academic researchers from tertiary institutions are required to publish their papers in journals that are indexed with the Web of Science (WoS) database. This key performance index is set by the Malaysian Research Assessment (MyRA) agency within the Ministry of Higher of Education [33], which monitors research universities’ performances and ratings. With the bulk of Malaysian research output coming from universities, it was plausible to postulate that academicians would have opted to publish their scientific works in international journals, as indexations of local journals in the WoS database were limited. Despite an increase in research outputs, progressive exposure and cultivation of research publications are needed to sustain the growing trend.

More than 60% of publications resulted from multicenter collaborations, with half being contributed by international partners (31.3%). This practice sees a positive growth of Malaysia’s scientific landscape, which thus reflects a recognition of the value of collaborative works among local scientists and researchers from different institutions. Our figure of international collaboration was higher than Cuba (22.9%) [30], Iran (21%) [12], and India (12.2%) [17]. International scientific collaborations bring greater impact and benefits to local researchers, such as transferable knowledge and skills from key experts, and exposure to state-of-the-art treatment, study designs, and technologies. It also increases dissemination channels, enabling publications in highly ranked journals, which allows for reaching out to international scientific communities [19,30].

From the distributional hotspot analyses, Kuala Lumpur was the most productive state as it houses the highest number of clinical academic institutions and researchers in the country. The discrepancy across all states across the country revealed opportunities to perform gap analyses before undertaking higher-impact research activities.

### 4.2. Citation Impact of Research Publications

Citation analyses provide a proxy measurement for the impact of research publications and research interest in a particular field. Furthermore, highly cited papers are identified as being excellent scientific works [34]. The number of citations for diabetes publications from Malaysia peaked in 2012, followed by a continuous deflection, which was less satisfactory, despite an increasing number of publications over the same period. A similar decreasing citation trend was also observed in another study [30]. There is a need to understand the declining trend of citations and potential associated factors, such as publication visibility and current research interest trends. A plausible explanation for such a phenomenon to occur may be attributed to the lead time bias for citing scholarly works. Such biases may accrue a higher number of citations for older manuscripts than recently published ones. The current study observed that papers published since 2012, and especially those from 2018, had somewhat less time to be cited than those published in or before 2012. With only 48 publications (2.3%) having more than 100 citations and up to one-fifth yet to be cited, efforts are required to make diabetes research in Malaysia to be more visible to the wider scientific community and create value from study findings to pave the way as references for future studies, both locally and internationally. Narin et al. [35] reported that international collaboration increases the publication citation by a factor of 2.5, whereas national collaboration increases it by a factor of 1.5 as compared to a publication from a single institution [35]. However, our study observed that publications without a collaboration topped the citation loads (37.8%), compared to those with international (37.6%) and national (24.6%) collaborations. Nine of the top 10 most-cited publications were dominated by academic institutions with the one from the Ministry of Health having received at least 210 citations.

### 4.3. Source Evaluation of Research Productivity

Diabetes-related research in Malaysia was published extensively in over 800 journals locally and worldwide. Academic researchers from public and private universities continue to drive the research output, similar to other studies [12,17,30]. The radar chart identified the top five key leaders in the field who were highly productive and contributed to the strongest collaborative link strengths. Strategies should be explored to enable more excellent publications in high-impact journals and evaluate the impact of research findings translated into policy or actions.

### 4.4. Content Evaluation of Research Outputs

We observed two distinct clusters of diabetes research output in Malaysia. The first cluster, clinical studies, involved persons with diabetes-related risk factors, glycemic control, and complications. The significant effort invested in preclinical studies that involved cellular and animal studies has led to the emergence of the second major cluster. Co-development of these clusters created windows of opportunities for a translational bench to develop future studies. We recorded limited evidence synthesizing results from randomized controlled trials and meta-analyses compared to China [18]. The eleven themes of the cluster concepts classified based on keywords provided insights into the focus across various aspects of diabetes using different study designs. Type 2 diabetes carried the highest weight of the total research outputs compared to type 1 and gestational diabetes, similar to other comparative studies [18,36].

### 4.5. Study Limitations and Strengths

This study did not include citation analyses in guidelines and/or policy documents. Detailed analyses of journal quartiles and self-citations were also beyond this study. The strength of this study was the application of comprehensive search strategies using three international databases and a local database to capture all publications, including those in non-indexed local journals. A useful topic to investigate for future studies would be to include citations from guidelines and policy documents, as these sources are most likely to be a direct measure of a publications’ impact on policies and practices. To correct for the lead time bias, future studies could analyze citation rates of scholarly works, that is the number of citations divided by the number of years since the publication [37]. However, this approach has its limitation and may result in under-representation of the entire scientific landscape [38]. Future works could adopt applications of more robust methodologies through machine learning approaches for the improved interpretability of huge datasets. These applications may be used to generate citation networks that infer key features for categorical classifications, such as the application of regularized simple graph convolution (SGC) [39] or network modeling that produces sequences of words and graphs [40]. Such techniques have the capacity to generate more powerful network visualizations for greater interpretability.

## 5. Conclusions

There were growing interests with collective efforts from the local scientific community that contributed to the growth of the diabetes research output over time. Nonetheless, given the diabetes burden in Malaysia, the cumulative evidence remained inadequate to guide policymaking to bend the epidemic curve in the population. Needs and gaps analyses that are supported by funding are required to reorient research priorities for matching the nation’s need to combat diabetes. It is crucial for Malaysia to nurture a diverse scientific workforce that would be capable to navigate from the conventional diabetes research priorities to a more robust and holistic research approach. Such efforts are crucial for producing real-time evidence to prevent, cure, and improve outcomes for people with or at risk for diabetes. Although clinical trials of numerous drugs are increasingly being conducted, we lack information to individualize therapy based on the population’s demographic, physiological, environmental, or genetic variations. Studies should also examine the durability of risk reduction, the cost-effectiveness of the interventions, and their potential impact to reduce diabetes complications. Population-based epidemiological studies that could identify potential risk within communities from different geographical areas through spatial variations and studies that could enhance the delivery of effective health promotion programs should be of utmost priority to accelerate diabetes prevention and control. With the evolvement of scientific enterprise to incorporate more robust research and statistical methodologies, it is important for Malaysia to approach its diabetes situation using contemporary basic science and translational, epidemiological, clinical, and omics research. These approaches should be integrated with computational methodologies through data science and have the capacity to synthesize real-time evidence for urgent policy and guidelines to be developed and adopted for practice.

## Figures and Tables

**Table 1:** Frequency of publications and citations based on collaborations.

Year	International Collaboration		National Collaboration		No Collaboration	
	Publication	Citation	Publication	Citation	Publication	Citation
2000	3	103	0	0	11	261
2001	0	0	1	54	4	58
2002	3	210	5	81	9	453
2003	2	110	1	19	10	249
2004	6	260	4	47	12	296
2005	5	354	9	145	20	532
2006	7	631	12	315	25	608
2007	14	676	14	298	28	846
2008	20	640	19	548	43	1008
2009	15	1269	20	345	42	1472
2010	32	1027	29	755	59	1673
2011	41	1324	30	955	58	996
2012	42	1827	42	830	82	1902
2013	61	1692	45	1134	83	1279
2014	61	840	68	1291	73	947
2015	67	885	104	1121	49	238
2016	69	732	80	524	52	321
2017	99	628	62	189	71	216
2018	108	167	61	46	102	70
Total	655	13,375	606	8697	833	13,425

**Table 2:** Top 10 most-cited papers by Malaysian authors.

No.	Title	Author	Year of Publication	Journal Name	Citations	MOH/University	Collaboration Type
1	Liraglutide, a once-daily human GLP-1 analogue, added to a sulphonylurea over 26 weeks produces greater improvements in glycaemic and weight control compared with adding rosiglitazone or placebo in subjects with Type 2 diabetes (LEAD-1 SU)	Marre, M.; Shaw, J.; Brandle, M.; Bebakar, W.M.W.; Kamaruddin, N.A.; Strand, J.; Zdravkovic, M.; Le Thi, T.D.; Colagiuri, S.	2009	*Diabetic Medicine*	947	USM	International Collaboration
2	How common is non-alcoholic fatty liver disease in the Asia-Pacific region and are there local differences?	Amarapurkar, D.N.; Hashimoto, E.; Lesmana, L.A.; Sollano, J.D.; Chen, P.J.; Goh, K.L.	2007	*Journal of Gastroenterology and Hepatology* (Australia)	387	UM	International Collaboration
3	The role of oxidative stress and antioxidants in diabetic complications	Matough, F.A.; Budin, S.B.; Hamid, Z.A.; Alwahaibi, N.; Mohamed, J.	2012	*Sultan Qaboos University Medical Journal*	324	UKM	International Collaboration
4	In vitro alpha-glucosidase and alpha-amylase enzyme inhibitory effects of *Andrographis paniculata* extract and andrographolide	Subramanian, R.; Asmawi, M.Z.; Sadikun, A.	2008	*Acta Biochim Pol*	274	USM	No Collaboration
5	Antidiabetic and antioxidant activity of *Annona squamosa* extract in streptozotocin-induced diabetic rats	Kaleem, M.; Asif, M.; Ahmed, Q.U.; Bano, B.	2006	*Singapore Medical Journal*	257	UIAM	International Collaboration
6	An alarmingly high prevalence of diabetic nephropathy in Asian type 2 diabetic patients: The MicroAlbuminuria Prevalence (MAP) Study	Wu, A.Y.T.; Kong, N.C.T.; De Leon, F.A.; Pan, C.Y.; Tai, T.Y.; Yeung, V.T.F.; Yoo, S.J.; Rouillon, A.; Weir, M.R.	2005	*Diabetologia*	243	UKM	International Collaboration
7	Omega-3 fatty acids: a comprehensive review of their role in health and disease	Yashodhara, B.M.; Umakanth, S.; Pappachan, J.M.; Bhat, S.K.; Kamath, R.; Choo, B.H.	2009	*Postgraduate Medical Journal*	241	Manipal Medical College	No Collaboration
8	Diabetes-associated macrovasculopathy: pathophysiology and pathogenesis	Rahman, S.; Rahman, T.; Ismail, A.A.; Rashid, A.R.	2007	*Diabetes, Obesity and Metabolism*	216	USM	No Collaboration
9	The improvement of hypertension by probiotics: effects on cholesterol, diabetes, renin, and phytoestrogens	Lye, H.S.; Kuan, C.Y.; Ewe, J.A.; Fung, W.Y.; Liong, M.T.	2009	*International Journal of Molecular Sciences*	210	USM	No Collaboration
10	Prevalence of diabetes in the Malaysian National Health Morbidity Survey III 2006	Letchuman, G.R.; Wan Nazaimoon, W.M.; Wan Mohamad, W.B.; Chandran, L.R.; Tee, G.H.; Jamaiyah, H.; Isa, M.R.; Zanariah, H.; Fatanah, I.; Ahmad Faudzi, Y.	2010	*Medical Journal of Malaysia*	204	MOH	National Collaboration

MOH = Ministry of Health, USM = Universiti Sains Malaysia, UM = Universiti Malaya, UKM = Universiti Kebangsaan Malaysia, UIAM = Universiti Islam Antarabangsa Malaysia.

**Table 3:** Top 10 sources of diabetes research output productivity in Malaysia.

No.	Journal	Total	Percentage
1	*Medical Journal of Malaysia*	95	4.5
2	*Institute of Electrical and Electronics Engineers (IEEE)*	68	3.2
3	*Diabetes Research and Clinical Practice*	45	2.1
4	*Singapore Medical Journal*	31	1.5
5	*International Medical Journal*	29	1.4
6	*Malaysian Journal of Medical Sciences*	27	1.3
7	*Evidence-Based Complementary and Alternative Medicine*	25	1.2
8	*PLoS One*	23	1.1
9	*International Medical Journal Malaysia*	22	1.1
10	*Tropical Journal of Pharmaceutical Research*	22	1.1

Note: A total of 807 journals accommodated publications of all diabetes research output in Malaysia (*n* = 2094) between 2000–2018.

**Table 4:** Frequent keywords and cluster conceptualization.

Cluster Size	Cluster Concept	Keywords	Thematic Interpretation
1 (75 items)	Clinical studies	Human (1141), male (947), female (813), adult (748), type 2 diabetes mellitus (745), diabetes mellitus (662), middle aged (501), major clinical study (457), age (430), hemoglobin a1c (319), risk factor (252), cross-sectional study (209), hypertension (199), glycemic control (174), body mass index (159), prevalence (158), type 1 diabetes mellitus (135), complication (153), treatment outcome (130), questionnaire (128), disease association (127), clinical article (105), retrospective study (102), disease duration (98), follow-up studies (94), nephropathy (90), prospective studies (86), quality of life (86), adolescent (83), risk assessment (80), case report (79), blood pressure (78), oral antidiabetic agent (78), outcome assessment (78), young adult (75), hemoglobin blood level (74), Asian (68), case-control study (68), cohort studies (63), comorbidity (63), aged 80 and over (62), creatinine (62), disease severity (62), primary healthcare (59), neuropathy (57), demography (56), genetics (56), diabetic foot (53), sex difference (53), clinical feature (52), mortality (51), incidence (50), diabetes control (46), disease course (46), exercise (46), waist circumference (46), wound healing (46), ethnology (45), time factor (45), age distribution (44), diet (43), genotype (42), antihypertensive agent (41), ethnicity (41), medical record (41), insulin treatment (40), physical activity (40), Chinese (39), Malaysian (39), creatinine blood level (38), ischemic heart disease (38), genetic association (37), lifestyle (37), smoking (37)	Clinical studies involving human subjects across different age groups. Some studies evaluated risks attributable to diabetes. These risks were evaluated across established demographic, anthropometric, and lifestyle factors, in addition to novel postulations, such as genetic associations. The cluster also explored diabetes complications, treatment efficacy, severity of the disease progression, and potential health and treatment outcomes using a variety of study designs and types, ranging from observational to interventional studies.
2 (45 items)	Animal studies	Controlled study (711), glucose blood level (464), animal model (449), glucose (421), nonhuman (421), insulin (397), Sprague Dawley rats (364), antidiabetic agent (332), diabetes (312), unclassified drug (244), streptozotocin-induced diabetes mellitus (229), metabolism (226), hypoglycemic agents (221), plant extract (199), drug effect (193), antioxidant (178), animal tissue (162), diabetes mellitus, experimental (149), hyperglycemia (125), body weight (114), glibenclamide (114), chemistry (109), animal cell (103), Wistar rats (92), insulin blood level (89), enzyme activity (77), histopathology (77), biological marker (72), medicinal plant (66), treatment duration (65), drug mechanism (63), plant leaf (63), dose response (58), enzyme inhibition (55), alpha glucosidase inhibitor (51), antidiabetic (48), mice (46), phytochemistry (45), flavonoid (43), drug dose–response relationship (42), anti-inflammatory agent (41), enzyme linked immunosorbent assay (38), phytotherapy (36), fruit (35), protein blood level (35)	This cluster emphasized laboratory-based experimental study design through animal models and cellular studies to explore potential novel drug therapies, ranging from chemical components to phytochemicals for the treatment of diabetes.
3 (9 items)	Co-morbidities	Low-density lipoprotein (129), triacylglycerol (125), high-density lipoprotein (125), cholesterol blood level (97), cholesterol (103), lipid (71), systolic blood pressure (56), diastolic blood pressure (41), blood sampling (35)	This cluster included studies that explored co-morbid conditions regarding diabetes, blood pressure, and lipid metabolism.
4 (9 items)	Drug studies	Randomized controlled trial (159), metformin (154), hypoglycemia (110), drug efficacy (104), drug safety (67), multicenter study (47), sulfonylurea (44), acarbose (40), monotherapy (36)	This cluster’s sole emphasis was on drug trials that evaluated the efficacy and safety of anti-diabetic therapies.
5 (8 items)	Biomarker studies	Oxidative stress (125), inflammation (56), superoxide dismutase (53), lipid peroxidation (43), malonaldehyde (41), catalase (39), interleukin 6 (36), C-reactive protein (35)	The cluster included studies that evaluated conventional and novel biomarkers.
6 (8 items)	Complications	Review (198), obesity (112), hyperlipidemia (106), cardiovascular disease (94), cardiovascular risk (57), metabolic syndrome (40), Asia (38), practice guideline (38)	This cluster included studies related to cardiovascular risk factors and macrovascular complications.
7 (8 items)	Treatment	Insulin resistance (100), pathophysiology (70), insulin release (65), gene expression (57), protein expression (55), pancreas islet beta-cell (50), insulin sensitivity (47), physiology (46)	This cluster explored studies related to physiological processes and genetic influences on glucose metabolism.
8 (8 items)	Control measures	Blood glucose (238), blood (187), comparative study (71), diet restriction (68), blood glucose monitoring (56), diagnosis (44), diet therapy (37), fasting (35)	This cluster examined studies related to the control and prevention of diabetes.
9 (6 items)	Eye complications	Retinopathy (162), pathology (89), procedures (79), eye protection (58), sensitivity and specificity (44), image processing (36)	This cluster focused solely on microvascular complications of diabetes, emphasizing ophthalmic complications and treatment procedures.
10 (6 items)	Health-related quality of life, knowledge, attitude, and practices	Psychology (51), patient compliance (49), attitude to health (48), self-care (45), health knowledge, attitudes, practice (40), patient education (35)	This cluster solely evaluated health outcomes from a psycho-socio-behavioral perspective, together with conventional knowledge, attitudes, and practice studies.
11 (5 items)	Gestational diabetes	Pregnancy (67), pregnancy diabetes mellitus (59), gestational diabetes mellitus (54), glucose tolerance test (52), oral glucose tolerance test (51)	This cluster uniquely evaluated studies related to gestational diabetes and its diagnosis.

## Data Availability

The data presented in this study are available in Table S1.

## Supplementary Materials

The following are available online at https://www.mdpi.com/1660-4601/18/1/318/s1, Table S1: Supplementary information of papers included.

## References

[1] International Diabetes Federation (2020). Diabetes Facts & Figures. https://www.idf.org/aboutdiabetes/what-is-diabetes/facts-figures.html#:~:text=In%202019%2C,low%2D%20and%20middle%2Dincome%20countries.

[2] Zhu Y., Zhang C. (2016). Prevalence of Gestational Diabetes and Risk of Progression to Type 2 Diabetes: A Global Perspective. Curr. Diabetes Rep..

[3] Mobasseri M., Shirmohammadi M., Amiri T., Vahed N., Hosseini Fard H., Ghojazadeh M. (2020). Prevalence and Incidence of Type 1 Diabetes in the World: A Systematic Review and Meta-Analysis. Health Promot. Perspect..

[4] International Diabetes Federation (2020). Diabetes in South-East Asia. https://www.idf.org/our-network/regions-members/south-east-asia/diabetes-in-sea.html.

[5] International Diabetes Federation (2020). Western Pacific Members. https://idf.org/our-network/regions-members/western-pacific/members/108-malaysia.html.

[6] Ganasegeran K., Hor C.P., Jamil M.F.A., Loh H.C., Noor J.M., Hamid N.A., Suppiah P.D., Manaf M.R.A., Ch’ng A.S.H., Looi I. (2020). A Systematic Review of the Economic Burden of Type 2 Diabetes in Malaysia. Int. J. Environ. Res. Public Health.

[7] Sharma S., Thomas V.J. (2008). Inter-Country R&D Efficiency Analysis: An Application of Data Envelopment Analysis. Scientometrics.

[8] Ivancheva L. (2008). Scientometrics Today: A Methodological Overview. COLLNET J. Scientometr. Inf. Manag..

[9] Dragos C.M., Dinu V., Pop C.M., Dabija D.C. (2014). Scientometric Approach of Productivity in Scholarly Economics and Business. Econ. Res. Ekon. Istraž..

[10] Correia A., Paredes H., Fonseca B. (2018). Scientometric Analysis of Scientific Publications in CSCW. Scientometrics.

[11] Barbosa S.D.J., Silveira M.S., Gasparini I. (2017). What Publications Metadata Tell Us about the Evolution of a Scientific Community: The Case of the Brazilian Human-Computer Interaction Conference Series. Scientometrics.

[12] Rasolabadi M., Khaledi S., Ardalan M., Kalhor M.M., Penjvini S., Gharib A. (2015). Diabetes Research in Iran: A Scientometric Analysis of Publications Output. Acta Inform. Med..

[13] Emami Z., Hariri N., Khamseh M.E., Nooshinfard F. (2018). Mapping Diabetes Research in Middle Eastern Countries During 2007–2013: A Scientometric Analysis. Med. J. Islam. Repub. Iran.

[14] Tran B.X., Nguyen L.H., Pham N.M., Vu H.T.T., Nguyen H.T., Phan D.H., Ha G.H., Pham H.Q., Nguyen T.P., Latkin C.A. (2020). Global Mapping of Interventions to Improve Quality of Life of People with Diabetes in 1990–2018. Int. J. Environ. Res. Public Health.

[15] Kucharavy D., Guio R.D. (2015). Application of Logistic Growth Curve. Procedia Eng..

[16] Bagley S.C., White H., Golomb B.A. (2001). Logistic Regression in the Medical Literature: Standards for Use and Reporting, with Particular Attention to One Medical Domain. J. Clin. Epidemiol..

[17] Waltman L., van Eck N.J., Noyons E.C.M. (2010). A Unified Approach to Mapping and Clustering of Bibliometric Networks. J. Inform..

[18] Corbin J., Strauss A. (2014). Basics of Qualitative Research: Techniques and Procedures for Developing Grounded Theory.

[19] Krishnamoorthy G., Ramakrishnan J.S.D. (2009). Bibliometric Analysis of Literature on Diabetes (1995–2004). Ann. Lib. Inf. Stud..

[20] Gupta B.M., Kaur H., Bala A. (2011). Mapping of Indian Diabetes Research during 1999–2008: A Scientometric Analysis of Publications Output. DESIDOC J. Lib. Inf. Technol..

[21] Zhao X., Guo L., Yuan M., He X., Lin Y., Gu C., Li Q., Zhao L., Tong X. (2016). Growing Trend of China’s Contribution to Global Diabetes Research: A Systematic Literature Review. Medicine.

[22] Sweileh W.M., Sa’ed H.Z., Al-Jabi S.W., Sawalha A.F. (2014). Bibliometric Analysis of Diabetes Mellitus Research Output from Middle Eastern Arab Countries During the Period (1996–2012). Scientometrics.

[23] Harande Y.I. (2011). Exploring the Literature of Diabetes in Nigeria: A Bibliometrics Study. Afr. J. Diabetes Med..

[24] Harande Y.I., Alhaji I.U. (2014). Basic Literature of Diabetes: A Bibliometrics Analysis of Three Countries in Different World Regions. J. Lib. Inf. Sci..

[25] Geaney F., Scutaru C., Kelly C., Glynn R.W., Perry I.J. (2015). Type 2 Diabetes Research Yield, 1951–2012: Bibliometrics Analysis and Density-Equalizing Mapping. PLoS ONE.

[26] Institute for Public Health (IPH), National Institutes of Health, Ministry of Health Malaysia (2008). The Third National Health and Morbidity Survey 2006 (NHMS III) 2006, Diabetes Mellitus. http://iku.moh.gov.my/images/IKU/Document/REPORT/2006/DiabetesMellitus.pdf.

[27] Institute for Public Health (IPH), National Institutes of Health, Ministry of Health Malaysia (2020). National Health and Morbidity Survey (NHMS) 2019: Vol. I: NCDs—Non-Communicable Diseases: Risk Factors and Other Health Problems. http://iku.moh.gov.my/images/IKU/Document/REPORT/NHMS2019/Report_NHMS2019-NCD_v2.pdf.

[28] Department of Public Health, Ministry of Health Malaysia (2016). National Strategic Plan for Non-Communicable Disease Medium Term Strategic Plan to Further Strengthen the NCD Prevention and Control Program in Malaysia (2016–2025). https://www2.moh.gov.my/moh/resources/Penerbitan/Rujukan/NCD/National%20Strategic%20Plan/FINAL_NSPNCD.pdf.

[29] DOSM (2020). Time Series Population Projection in Malaysia by 2025. https://www.data.gov.my/data/en_US/dataset/time-series-population-projection-by-ethnic-group-and-sex-malaysia-2020-2025-2030-2035-and-2040/resource/e47083dc-78a8-4ec2-872e-eb85e422c1f2.

[30] Corrales-Reyes I.E., Fornaris-Cedeño Y., Dorta-Contreras A.J., Mejia C.R., Pacheco-Mendoza J., Arencibia-Jorge R. (2019). Cuban Scientific Production on Diabetes, 2000–2017: Peer-reviewed Publications, Collaboration and Impact. MEDICC Rev..

[31] Hussein Z., Taher S.W., Gilcharan Singh H.K., Chee Siew Swee W. (2015). Diabetes Care in Malaysia: Problems, New Models, and Solutions. Ann. Glob. Health.

[32] Ganasegeran K., Ch’ng A.S.H., Jamil M.F.A., Looi I. (2020). Clinicians’ Publication Output: Self-Report Survey and Bibliometric Analysis. Publications.

[33] Ministry of Higher Education (2020). Malaysia Research Assessment (MyRA). https://www.mohe.gov.my/en/.

[34] Aksnes D.W. (2003). Characteristics of Highly Cited Papers. Res. Eval..

[35] Narin F., Stevens K., Whitlow E. (2005). Scientific Co-Operation in Europe and the Citation of Multinationally Authored Papers. Scientometrics.

[36] Bala A., Gupta B.M. (2012). Diabetes Research in India, China and Brazil: A Comparative Quantitative Study, 2000–2009. J. Health Med. Inform..

[37] Hughes D., Hughes A., Powell A., Al-Sarireh B. (2019). Hepatocellular Carcinoma’s 100 Most Influential Manuscripts: A Bibliometric Analysis. Int. J. Hepatobiliary Pancreat. Dis..

[38] Powell A.G., Hughes D.L., Wheat J.R., Lewis W.G. (2016). The 100 Most Influential Manuscripts in Gastric Cancer: A Bibliometric Analysis. Int. J. Surg..

[39] Pho P., Mantzaris A.V. (2020). Regularized Simple Graph Convolution (SGC) for Improved Interpretability of Large Datasets. J. Big Data.

[40] Perozzi B., Al-Rfou R., Skiena S. DeepWalk—Online Learning of Social Representations. Proceedings of the 20th ACM SIGKDD International Conference on Knowledge Discovery and Data Mining.

